# Using PROMIS-29 to determine symptom burdens in the context of the Type 1 and 2 systemic lupus erythematosus (SLE) model: a cross sectional study

**DOI:** 10.1186/s41687-023-00678-5

**Published:** 2023-12-21

**Authors:** Kai Sun, Amanda M. Eudy, Nathaniel Harris, David S. Pisetsky, Lisa G. Criscione-Schreiber, Rebecca E. Sadun, Jayanth Doss, Megan E. B. Clowse, Jennifer L. Rogers

**Affiliations:** 1 Division of Rheumatology and Immunology, Department of Medicine, Duke University School of Medicine, DUMC 2978, Durham, NC 27710 USA; 2 Durham VA Medical Center, Durham, NC USA

**Keywords:** Systemic lupus erythematosus, Patient-reported outcomes, Health-related quality of life

## Abstract

**Objective:**

To account for heterogeneity in systemic lupus erythematosus (SLE) and bridge discrepancies between patient- and physician-perceived SLE activity, we developed the Type 1 and 2 SLE model. We examined PROMIS-29 scores, a composite patient-reported outcome (PRO) measure, through the lens of the model.

**Methods:**

Patients completed PROMIS-29 and the polysymptomatic distress scale (PSD). Rheumatologists completed the SLE disease activity index (SLEDAI), and physician’s global assessments (PGAs) for Type 1 and 2 SLE. We defined Type 1 SLE using SLEDAI, Type 1 PGA, and active nephritis, and Type 2 SLE using PSD and Type 2 PGA. We compared PROMIS-29 T-scores among Type 1 and 2 SLE groups and explored whether PROMIS-29 can predict Type 1 and 2 SLE activity.

**Results:**

Compared to the general population, patients with isolated Type 1 SLE reported greater pain and physical dysfunction but less depression and improved social functions; patients with high Type 2 SLE (irrespective of Type 1 activity) reported high levels of pain, fatigue, and social and physical limitations. Patients with minimal Type 1 and 2 SLE had less depression and greater physical functioning with other domains similar to national norms. PROMIS-29 predicted Type 2 but not Type 1 SLE activity.

**Conclusion:**

PROMIS-29 similarities in patients with high Type 2 SLE, with and without active Type 1 SLE, demonstrate the challenges of using PROs to assess SLE inflammation. In conjunction with the Type 1 and 2 SLE model, however, PROMIS-29 identified distinct symptom patterns, suggesting that the model may help clinicians interpret PROs.

## Introduction

Systemic lupus erythematosus (SLE) is a multi-organ autoimmune disease. People living with SLE experience a broad array of symptoms with a range of intensities that often vary over time. The heterogeneity of SLE makes the disease not only challenging to manage but its outcomes difficult to measure [1].

Recent efforts to improve clinical assessments have focused on incorporating patients’ perspective through the use of patient-reported outcomes (PROs) in clinical trials and research studies, such as the patient-reported outcomes measurement information system (PROMIS) developed by the National Institutes of Health [2–4]. PROMIS instruments have the ability to assess and compare patient-perceived health status across clinically diverse populations [5]. PROMIS domains have also been identified as relevant to SLE and correlated with other patient-reported measures of SLE symptom severity and declining health status [6–8].

A key challenge for using PROMIS and other PROs in SLE, however, is that they measure the severity of symptoms, but do not determine the symptoms’ etiology [1]. In fact, PROMIS scores do not correlate strongly with physician-reported measures of SLE activity or damage when lupus is considered a single clinical condition [7, 9, 10]. This gap between patient- and physician-reported measures of SLE activity has been well described and reflect PROMIS’ limited utility in determining the degree of inflammatory activity in this disease [11, 12]. The gap between patient- and physician-perceived SLE activity, if not carefully accounted for, can also contribute to patient-physician miscommunication and mistrust [13–15].

In an effort to both account for the heterogeneity inherent to SLE and bridge the discrepancy between patient- and physician-perceived SLE activity, our group developed the Type 1 and 2 SLE model for symptom categorization [16]. The Type 1 and 2 SLE model is intended to encompass the full spectrum of symptoms and manifestations experienced by patients and promote a more comprehensive and holistic approach to SLE assessment and management [16, 17].

In our model, Type 1 SLE manifestations represent the classic signs and symptoms of SLE that have clear links to immune disturbance. These manifestations include arthritis, nephritis, and pericarditis and are typically assessed by the SLEDAI (SLE disease activity index) [18], BILAG (British Isles Lupus Assessment Group) [19], or other validated measures of disease activity.

In contrast to Type 1 manifestations, Type 2 SLE manifestations include symptoms such as fatigue, widespread pain, and mood and cognitive disturbances, whose origin is unclear. Based on qualitative interviews with a representative sample of people with SLE, we hypothesize that Type 2 symptoms have distinct patterns and causes. During some periods of the SLE disease course, Type 2 symptoms may be due to active inflammation related to Type 1 SLE. At other times, they may be caused by a range of etiologies, including SLE damage, sleep disorders, medication effects, psychosocial distress, and many others [20, 21]. While Type 2 SLE symptoms appear to be responsible for much of patients’ perception of disease activity [17, 22], these symptoms are generally not captured by existing SLE disease activity measures [16, 21].

While prior SLE studies have found overall moderate limitations in quality of life (QoL) using the PROMIS measures, we assessed whether over-laying the construct of the Type 1 and 2 SLE Model could identify more specific patterns of limitations in QoL. Doing so can potentially identify particular domains of quality of life that may benefit from interventions within sub-groups of patients. We also sought to assess the utility of PROMIS in identifying patients with substantial Type 2 SLE symptoms in an effort to begin to bridge the gap between patient and physician perceptions of SLE disease activity.

## Patients and methods

### Study population

Patients with SLE who met the 1997 American College of Rheumatology (ACR) [23] or 2012 Systemic Lupus International Collaborating Clinics (SLICC) classification criteria [24] were invited to enroll in the Duke Lupus Registry (DLR), a prospective registry of patients who receive care at the Duke University Lupus Clinic. Over 90% of patients invited agree to participate in DLR. Eligible patients were at least 18 years of age, fluent in English, and provided informed consent. This study included consecutive patients in the DLR and was approved by Duke Health IRB (Pro00008875, Pro00108618).

### Data collection

At study entry, patients self-reported socio-demographic information. At each clinic visit, patients’ treating rheumatologist completed disease activity measures, including the SLE Disease Activity Index (SLEDAI), Physician’s Global Assessment of Disease Activity (PGA) for Type 1 SLE activity, and a PGA for Type 2 SLE activity [25–27]. Both Type 1 and Type 2 PGAs were scored on a 4-point scale (0–3) with 0 indicating no activity, 1 mild activity, 2 moderate activity, and 3 severe activity. Additionally, the following patient reported outcomes were collected:

*PROMIS-29* is a 29-question, disease-agnostic survey that includes 4-item scales measuring 7 domains (physical function, pain interference, fatigue, sleep disturbance, ability to participation in social roles, depression, and anxiety). Raw scores were uploaded to the scoring service, where T-scores were obtained [28]. A T-score of 50 correlates to the reference population mean, with standard deviation of 10. A 5-point difference (half standard deviation) is considered a clinically significant difference [29, 30]. Higher scores indicate more of the domain.

*Polysymptomatic distress (PSD) scale* allows for assessment of Type 2 SLE-associated symptoms on a continuum. The scale is obtained by summing the two components of the 2016 ACR Fibromyalgia Criteria, the Widespread Pain Index and Symptom Severity Scale, with scores ranging from 0 to 31 [31, 32]. A total PSD score of ≥ 8 is considered moderate to severe polysymptomatic distress [33].

### Type 1 and 2 SLE classification groups

High Type 1 SLE was defined as SLEDAI ≥ 6, Clinical SLEDAI (SLEDAI scored without serologic descriptors) [34] ≥ 4, active nephritis (i.e., evidence of proteinuria, pyuria, or hematuria by SLEDAI criteria), or Type 1 PGA ≥ 1. High Type 2 SLE was defined as PSD ≥ 8 or Type 2 PGA ≥ 1. Patients with both low Type 1 and 2 SLE symptoms were classified as Minimal SLE, and inversely, patients with both high Type 1 and 2 SLE symptoms were classified as Mixed SLE [20]. The Type 1 and 2 SLE classification and PROMIS scores from the same clinic visit were used in this analysis.

### Statistics

In this cross-sectional analysis, categorical variables were described with percentages, and continuous variables were summarized with either mean (standard deviation) or median (interquartile range), depending upon distribution. We compared PROMIS T-scores across Type 1 and 2 SLE classification groups using the Kruskal–Wallis test. We performed a second analysis comparing the proportion of patients with T-scores 5 points (half a standard deviation) worse than the population mean (score ≤ 45 for physical and social functions, and ≥ 55 for the rest of the domains), with differences across groups estimated by Fisher’s exact test.

Logistic regression models determined the ability of PROMIS-29 measures to predict Type 1 and Type 2 SLE activity. Using all PROMIS-29 domains as independent variables, we separately estimated predicted probabilities of high Type 1 SLE and high Type 2 SLE for each patient. We compared the models’ predicted probabilities with our study definitions for high Type 1 SLE and high Type 2 SLE, and then estimated the proportion of patients with correctly predicted Type 1 and Type 2 SLE activity. We then estimated the hit rate, chance hit rate, and Huberty’s *I* index, with a Huberty’s *I* index > 0.35 indicating the models’ ability to predict high Type 1 SLE and high Type 2 SLE activity over chance [35]. To identify the most parsimonious model, backward selection determined independent predictors of high Type 1 and high Type 2 SLE, separately, with predictors retained if α < 0.05.

Statistical analysis was performed using SAS 9.4.

## Results

We included 120 patients in this analysis. The median age was 43 years, 95% were female, and 57% were Black. Twelve patients (10%) were categorized as having only Type 1 SLE activity at their visit, 31 (26%) as having only Type 2 SLE activity, 34 (28%) had high Type 1 and 2 SLE activity (Mixed SLE), and 43 (36%) had low Type 1 and 2 SLE activity (Minimal SLE). Patients in the Type 1 SLE group were more likely to be Black (92%), and patients with Type 2 SLE activity (Type 2 alone or Mixed) had higher rates of medical disability. Fourteen percent of participants met 2016 ACR criteria for fibromyalgia [31] on the day of the study visit (Table 1).

**Table 1:** Socio-demographic and clinical characteristics of patients included in the study

Patient characteristics	Minimal (n = 43)	Type 1 (n = 12)	Type 2 (n = 31)	Mixed (n = 34)	*p* value
Socio-demographics, N (%)					
Black	24 (56%)	11 (92%)	14 (45%)	19 (56%)	0.04
Female	42 (98%)	11 (92%)	31 (100%)	30 (88%)	0.08
Medically disabled	10 (23%)	4 (33%)	14 (45%)	19 (56%)	0.03
Employed	23 (53%)	6 (55%)	13 (43%)	13 (35%)	0.4
Medicaid insurance	3 (7%)	4 (33%)	7 (23%)	7 (21%)	0.07
Married	23 (53%)	3 (25%)	14 (45%)	14 (41%)	0.3
≥ College education	27 (63%)	5 (45%)	16 (52%)	16 (47%)	0.5
Clinical factors, median (IQR)					
Age	44 (34–52)	35 (29–46)	46 (35–57)	41 (33–50)	0.3
Years since diagnosis	15 (7–22)	16 (12–28)	15 (8–21)	14 (9–20)	0.6
Measures of Type 1 SLE activity, median (IQR)					
SLEDAI	0 (0–2)	7 (5–10)	0 (0–2)	6 (4–8)	< 0.0001
Clinical SLEDAI	0 (0–0)	3 (0–4)	0 (0–0)	4 (2–6)	< 0.0001
Type 1 PGA	0 (0–0.2)	1 (0.5–1.5)	0 (0–0.5)	1 (0.5–1.5)	< 0.0001
Active nephritis	0 (0%)	5 (42%)	0 (0%)	8 (23%)	< 0.0001
Measures of Type 2 SLE activity, median (IQR)					
PSD score	4 (1–5)	5 (2–6)	10 (8–14)	11 (8–18)	< 0.0001
Type 2 PGA	0 (0–0.2)	0 (0–0.5)	1 (0.5–2)	1 (1–2)	< 0.0001
Fulfilled FM criteria	0%	0%	13%	35%	< 0.0001

*IQR* interquartile range, *PGA* physician global assessment, range 0–3, *PSD* polysymptomatic distress, range 0–31, *SLEDAI* systemic lupus erythematosus disease activity index, range 0–105, *FM* fibromyalgia, according to the 2016 ACR Criteria [31]

### Overall

Compared to the US general population mean T-score of 50, our overall cohort of patients with SLE had clinically significantly worse scores in fatigue, pain interference, sleep disturbance, and physical function. They had similar scores to the general population in social function, anxiety, and depression (Table 2).

**Table 2:** PROMIS-29 T-scores by Type 1 and 2 SLE categorization

PROMIS domains, median (IQR)	Overall (n = 120)	Minimal (n = 43)	Type 1 (n = 12)	Type 2 (n = 31)	Mixed (n = 34)	*p* value
Depression	*41*^a^* (41–54)*	*41 (41–52)*	*41 (41–45)*	**46 (41–54)**	**51 (41–56)**	0.05
Anxiety	**51 (40–58)**	**48 (40–56)**	**46 (40–56)**	**54 (40–60)**	**53 (49–60)**	0.04
Social Function	**50 (42–58)**	**52 (50–64)**	*55 (48–61)*	44 (39–50)	44 (40–52)	< 0.0001
Fatigue	56 (49–64)	**49 (43–53)**	**50 (34–55)**	***63 (57–69)***	***63 (57–70)***	< 0.0001
Sleep Disturbance	56 (54–59)	**54 (51–58)**	57 (56–59)	57 (54–61)	56 (54–59)	0.03
Pain interference	56 (50–61)	**52 (42–56)**	56 (42–59)	***61 (56–67)***	***61 (56–67)***	< 0.0001
Physical Function	42 (37–57)	*57 (43–57)*	43 (39–47)	***39 (34–43)***	***39 (35–43)***	< 0.0001

PROMIS T-score means are 50 for the reference population, with a difference of 5 (half standard deviation) representing a clinically significant difference [29, 30]. A higher score indicates more of the domain being measured. Depression, anxiety, sleep disturbance, fatigue, and pain interference: higher score indicates worse problem. Social and physical functioning: lower score indicates worse problem. *p* values are generated using the Kruskal–Wallis test to compare PROMIS T-scores across Type 1 and 2 SLE categorization groups.

^a^The mean Depression T-score was 47.8 (SD 8.4) with more than half of the participants scoring 40–45

Italic: Better than general population (T-score ≥ 5 points better than mean of 50)

Bold: Same as general population (T-score between 45 and 55)

Underline: Worse than general population (T-score ≥ 5 but < 10 points worse than mean of 50)

Bold and italic: Much worse than general population (≥ 10 points worse than mean of 50)

*PROMIS* patient-reported outcome measurement information system, *SRA* social roles and activities

### Minimal SLE

**Fig. 1:**
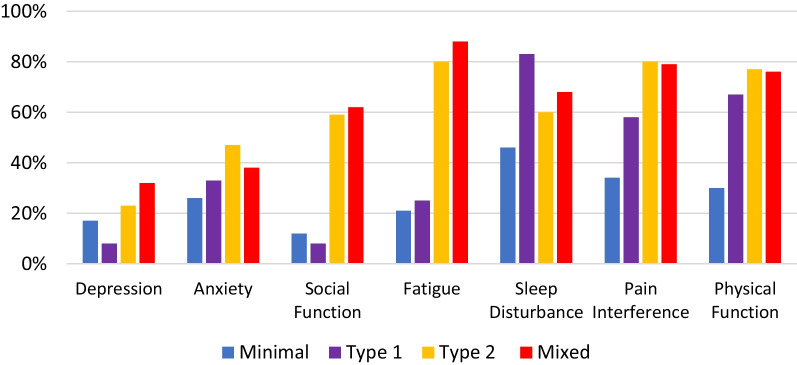
Proportion of patients with PROMIS T-scores half standard deviation worse than the reference population mean

Patients with Minimal SLE had PROMIS T-scores similar to the US general population mean in all domains except they had less depression and greater physical function (Table 2). While 46% reported clinically significant sleep disturbance, only 21% reported fatigue (Fig. 1).

### Type 1 SLE

When compared with the US general population, patients with isolated Type 1 SLE did not have anxiety, depression, or limitations in social functioning. On the other hand, they reported clinically significant higher pain interference and limitations in physical functioning (Table 2). While 83% reported clinically significant sleep disturbance, only 25% reported fatigue (Fig. 1).

### Type 2 and mixed SLE

Patients with high Type 2 SLE symptoms (irrespective of Type 1 SLE activity) reported a similar pattern of PROMIS scores. Patients in both the Type 2 and Mixed groups reported high levels of physical and social dysfunction as well as clinically significant levels of sleep disturbance and fatigue. Neither group, however, reported clinically significant levels of anxiety or depression (Table 2).

### Comparing across groups

There were significant differences across the four groups of patients in social function, fatigue, pain interference, depression and physical functioning. The four groups were not significantly different in the level of anxiety or sleep disturbance. The largest numeric differences between patients with and without high Type 2 symptoms were in social functioning and fatigue. Sleep disturbance was common in all groups, but fatigue was only increased in patients with high Type 2 symptoms. Table 3 summarizes characteristics of the Type 1 and 2 SLE groups based on PROMIS-29 and physician-reported Type 1 SLE activity.

**Table 3:** Characteristics of Type 1 and 2 SLE activity groups

Type 1 and 2 SLE classification	PROMIS	Physician-reported Type 1 SLE activity
Minimal SLE	No limitations	Low
Type 1 SLE	Moderate pain and physical dysfunction	High
	Sleep disturbance	
Type 2 SLE	Severe pain and physical dysfunction	Low
	Fatigue and sleep disturbance	
	Social dysfunction	
Mixed SLE	Severe pain and physical dysfunction	High
	Fatigue and sleep disturbance	
	Social dysfunction	

### Using PROMIS-29 to identify patients with current Type 2 SLE activity

Results from logistic regression models showed that the model accurately predicted high Type 2 SLE activity, with a hit rate of 83% and Huberty’s *I* index of 0.66, indicating the model was able to classify patients’ Type 2 SLE activity over chance (Table 4). Backward regression models identified fatigue and physical function as significant predictors of high Type 2 SLE activity. Inclusion of only these two domains in the logistic regression model still allowed for the accurate prediction of high Type 2 SLE activity, with a hit rate of 82%. In contrast, the logistic regression model accurately predicted high Type 1 SLE activity in 61% of patients, with a Huberty’s *I* index of 0.18, indicating an inability of the PROMIS-29 measures to classify patients’ Type 1 SLE activity.

**Table 4:** Utility of PROMIS-29 domains to identify patients with high Type 1 and high Type 2 SLE activity

	Type 1 SLE		Type 2 SLE	
	All domains	Backward selection	All domains	Backward selection
	OR (95% CI)	OR (95% CI)	OR (95% CI)	OR (95% CI)
Depression	1.02 (0.95, 1.09)	–	1.03 (0.84, 1.13)	–
Anxiety	1.00 (0.94, 1.07)	–	0.99 (0.90, 1.09)	–
Social function	1.04 (0.97, 1.11)	–	1.02 (0.93, 1.11)	–
Fatigue	1.02 (0.97, 1.07)	–	1.23 (1.11, 1.35)	1.22 (1.12, 1.32)
Sleep disturbance	1.03 (0.95, 1.12)	–	1.00 (0.87, 1.15)	–
Pain interference	1.01 (0.94, 1.08)	–	1.00 (0.91, 1.09)	–
Physical function	0.92 (0.85, 1.00)	0.93 (0.89, 0.97)	0.91 (0.83, 1.00)	0.92 (0.86, 0.98)
Hit rate	61%	59%	83%	82%
Chance hit rate	52%	52%	51%	51%
Huberty’s *I* index	0.18	0.15	0.66	0.63

High type 1 SLE was defined as SLE disease activity index (SLEDAI) ≥ 6, Clinical SLEDAI (SLEDAI scored without serologic descriptors) ≥ 4, active nephritis (i.e., evidence of proteinuria, pyuria, or hematuria by SLEDAI criteria), or Type 1 physician global assessment (PGA) ≥ 1; High Type 2 SLE was defined as polysymptomatic distress scale (PSD) ≥ 8 or Type 2 PGA ≥ 1

## Discussion

The results of this study showed that although PROMIS-29 scores of our overall cohort were similar to prior studies, dividing patients based on their current Type 1 and 2 SLE symptoms allowed us to identify more homogeneous groups with distinct patterns in quality of life [36–38]. As our data indicate, when compared to the general population, patients with Minimal SLE symptoms reported similar or better quality of life; patients with isolated current Type 1 SLE had superior mental and social health but more pain and limitations in physical functioning; and patients with current Type 2 symptoms (regardless of whether their Type 1 SLE was active) reported high levels of fatigue, pain, and limitations in social and physical functioning. Additionally, we found that the PROMIS-29 score patterns can effectively identify current Type 2 SLE activity, but not Type 1 SLE activity.

We believe that the inability of PROs to distinguish between patients with active inflammation (Mixed SLE) and those who do not (Type 2 SLE) can present a challenge to incorporating PROs within routine SLE care. Prior studies have demonstrated a discordance between patient- and provider-measures of SLE [11, 12]. Despite patients’ enthusiasm for using PROMIS measures within their rheumatic care, they, like rheumatologists, also describe challenges in discerning whether the symptoms reported in the measures are due to SLE-related inflammation or other conditions [1]. Understanding patient-reported symptoms in the context of the Type 1 and 2 SLE Model can help enhance physicians’ ability to interpret PROs [39]. Based on our data, PROs largely assess the extent of Type 2 SLE symptoms, and likely should not be used as a surrogate for Type 1 SLE activity. The Type 1 and 2 SLE Model allows PROs to no longer pit patients’ lived experience against physicians’ medical expertise but rather allow them to be incorporated into a comprehensive assessment of SLE.

The severe limitations in quality of life we observed in patients with Type 2 SLE were similar to people with fibromyalgia without an underlying autoimmune disease as demonstrated in the 2017 study by Katz et al. [36] Unlike patients with fibromyalgia, however, our patients have all met SLE classification criteria, and more than half of those with active Type 2 SLE also have objective findings of Type 1 SLE activity. Whereas the prevalence of fibromyalgia is 1–2% in the general population [40–42], the increase in fibromyalgia-like Type 2 symptoms in SLE is striking − 14% of patients in this study met criteria for fibromyalgia, and widespread pain can affect 20–60% of patients with SLE [43–45]. Further, the presence of Type 2 SLE symptoms in patients with active Type 1 SLE raises the possibility that these symptoms may, in some patients and at some time points in the disease course, be caused by inflammation.

Besides inflammation, mood and sleep disturbance have been posited as other causes of Type 2 SLE symptoms. However, we found that mood disturbance in this cohort is not elevated compared to the general population, similar to prior studies in SLE using PROMIS measures [8, 37, 38, 46]. This is true even among patients with high Type 2 SLE. Nevertheless, other studies using depression measures (such as the Beck Depression Index) that include somatic symptoms suggest greater rates of depression among patients with SLE around 30–40% [47, 48]. The difference can be explained by the fact that the PROMIS depression scales do not include somatic symptoms such as fatigue which could be caused by SLE, anemia, or a range of other co-morbid conditions [46]. Similarly, we did not find a clear link between sleep disturbance and fatigue across the Type 1 and 2 SLE groups. In fact, sleep disturbance scores were elevated across all groups, but only half of the patients reported high levels of fatigue. Taken together, these findings suggest that Type 2 SLE symptoms are likely caused by a range of etiologies that require a tailored approach to each patient.

While we have previously used the PSD and physician global assessment to identify patients with current Type 2 activity, our data suggest that the PROMIS-29 as a whole and the PROMIS domains of fatigue and physical functioning can also be used. A prior study by Arcani et al. [49] used the SF-36 to categorize patients into those with and without active Type 2 symptoms. These findings suggest that this categorization system is robust and that a variety of patient-reported measures can be used as long as they assess key symptoms of Type 2 SLE [39].

Strengths of our study include the high representation of Black patients and the comprehensive patient- and provider-reported measures and clinical dataset with minimal missing data across our panel. While the analysis is limited to a relatively small sample size, we still detected significant differences in PROMIS scores across multiple domains. We are unable to make causal inferences due to the cross-sectional nature of the data and acknowledge that cross-sectional data are unable to capture fluctuations in patients’ symptoms over time. Further, because this study was conducted at a single tertiary lupus center in the US Southeast, our cohort may not be representative of patients with SLE in other clinical contexts or locations. Lastly, we were not able to assess cognitive dysfunction, another prominent feature of Type 2 SLE, in this study, and it should be evaluated in future work of Type 2 SLE.

In conclusion, we analyzed PROMIS-29 scores in patients with SLE through the lens of the Type 1 and 2 SLE Model and identified distinct patterns of PRO outcomes based on the current level of Type 1 and 2 SLE symptoms. Our data demonstrate the usefulness of the PROMIS-29 measures in identifying patients with active Type 2 SLE and the challenges of using these scores to identify active, inflammatory SLE. Further study is needed to assess the utility of the Type 1 and 2 SLE Model in identifying the distinct pathophysiologic causes of Type 2 SLE activity and enabling effective, patient-specific treatments to improve QoL. This study suggests that comprehensive patient-reported measures can be a useful tool in this work.

## Data Availability

Data are available upon reasonable request.

## Abbreviations

ACR: American College of Rheumatology
DLR: Duke Lupus Registry
FM: Fibromyalgia
IQR: Interquartile range
PGA: Physician global assessment
PRO: Patient-reported outcome
PROMIS: Patient-reported outcome measurement information system
PSD: Polysymptomatic distress
QoL: Quality of life
SLE: Systemic lupus erythematosus
SLICC: Systemic Lupus International Collaborating Clinics
SLEDAI: Systemic lupus erythematosus disease activity index
SRA: Social roles and activities
